# The Role of Glutathione in the Management of Cell-Mediated Immune Responses in Individuals with HIV

**DOI:** 10.3390/ijms25052952

**Published:** 2024-03-03

**Authors:** Nicole Lin, Thomas Erdos, Carson Louie, Raina Desai, Naomi Lin, Gregory Ayzenberg, Vishwanath Venketaraman

**Affiliations:** 1 College of Osteopathic Medicine of the Pacific, Western University of Health Sciences, Pomona, CA 91766, USAthomas.erdos@westernu.edu (T.E.); carson.louie@westernu.edu (C.L.); raina.desai@westernu.edu (R.D.); gregory.ayzenberg@westernu.edu (G.A.); 2 Creighton University School of Medicine, Creighton University, Omaha, NE 68178, USA; naomilin@creighton.edu

**Keywords:** antioxidants, cell-mediated immunity, free radicals, HIV, HIV-associated neurodegenerative diseases, interleukins, N-acetylcysteine, T cells

## Abstract

Human immunodeficiency virus (HIV) is a major cause of death worldwide. Without appropriate antiretroviral therapy, the infection can develop into acquired immunodeficiency syndrome (AIDS). AIDS leads to the dysregulation of cell-mediated immunity resulting in increased susceptibility to opportunistic infections and excessive amounts of inflammatory cytokines. HIV-positive individuals also demonstrate diminished glutathione (GSH) levels which allows for increased viral replication and increased pro-inflammatory cytokine release, further contributing to the high rates of mortality seen in patients with HIV. Adequate GSH supplementation has reduced inflammation and slowed the decline of CD4+ T cell counts in HIV-positive individuals. We aim to review the current literature regarding the role of GSH in cell-mediated immune responses in individuals with HIV- and AIDS-defining illnesses.

## 1. Introduction

The pathogenesis of HIV infection is intricately linked to chronic inflammation and oxidative stress, profoundly impacting the host immune system, and contributing to the progression of disease. During initial HIV infection, local tissue inflammation is triggered by the activation of innate immune cells (e.g., dendritic cells and macrophages), setting off a cascade of immune responses [1]. The release of pro-inflammatory cytokines such as IL-1, IL-6, TNF-α, and IL-17 leads to increased immune-cell activation and recruitment, creating an environment conducive to viral replication [2]. Paradoxically, these cytokines play pivotal roles in orchestrating immune responses against HIV but also contribute to the pathogenesis of the disease. Additionally, the activation of adaptive immune responses, including CD4+ and CD8+ T cells, further amplifies the inflammatory cascade [3]. Over time, this sustained immune activation results in chronic inflammation, which not only contributes to tissue damage but also fuels viral proliferation by providing a continuous supply of target cells, resulting in the gradual depletion of CD4+ T cells, hallmarking HIV progression. Furthermore, the sustained release of pro-inflammatory mediators induces oxidative stress, further contributing to immune dysfunction and HIV pathogenicity [4]. Thus, the complex interplay between chronic inflammation, oxidative stress, and dysregulated cytokine production underlies the progressive immune system deterioration observed in HIV infection. Understanding these mechanisms is critical for developing targeted therapeutic interventions to mitigate immune dysregulation and improve clinical outcomes in patients with HIV/AIDS.

Glutathione is the most abundant intracellular antioxidant, serving a critical role in redox homeostasis and protecting cells from oxidative stress by neutralizing reactive oxygen species [5]. In the context of the immune system, optimal glutathione levels are essential for the maintenance of a redox-balanced environment for the proper functioning of lymphocytes and macrophages [6,7]. Previous studies have shown that glutathione supplementation can modulate the cellular redox status for an improved immune response against infectious pathogens, notably Mycobacterium tuberculosis [8,9]. Deficiencies in glutathione have been associated with impaired immune function and increased susceptibility to various diseases, particularly in Individuals with HIV [10]. Therefore, maintaining adequate levels of glutathione is vital for supporting a robust immune system and preventing diseases linked to oxidative stress. The observed improvements in glutathione status and immune function in response to supplementation underscore its potential therapeutic value in managing infections and enhancing immune responses. These findings collectively accentuate the critical role of glutathione in modulating immune functions and suggest its potential as an adjunctive therapeutic strategy in infectious diseases, warranting further exploration in clinical settings.

## 2. Materials and Methods

This article investigates the role of glutathione in the pathophysiology and treatment of HIV and HIV-associated diseases. The literature involved in this review was mainly acquired by the use of the following databases: Pubmed, NCBI databases, and Google scholar. Terminology used to develop this topic mainly included “HIV”, “Glutathione”, “N-acetylcysteine”, “antioxidants”, “cell-mediated immunity”, “HIV-associated neurodegenerative diseases”, “interleukins”, “free radicals”, and “T cells”. The research articles selected for this review were chosen based on their significance and the credibility of their research design. Resources included random control studies, case–control studies, literature reviews, meta-analysis, and more. Articles were excluded due to old publication date, non-relevance, and poor effect size. A total of 71 articles were included in qualitative synthesis. 

## 3. Overview of Cell-Mediated Immunity

When our body encounters a pathogen, the body responds first with innate immunity and then with adaptive immunity, which includes humoral and cell-mediated immunity. Cell-mediated immunity refers to all immune responses that are not antibody-driven (such as the activation of macrophages, the activation of natural killer cells, and cytokines that respond to antigens rather than antibodies). Cell-mediated immunity involves the production of CD4+ T cells (which trigger antibody release from B cells and regulate other immune cells) and cytotoxic CD8+ T cells (which kill infected host cells). Some of the cells regulated by CD4+ T cells are macrophages and CD8+ T cells. 

**Figure 1:**
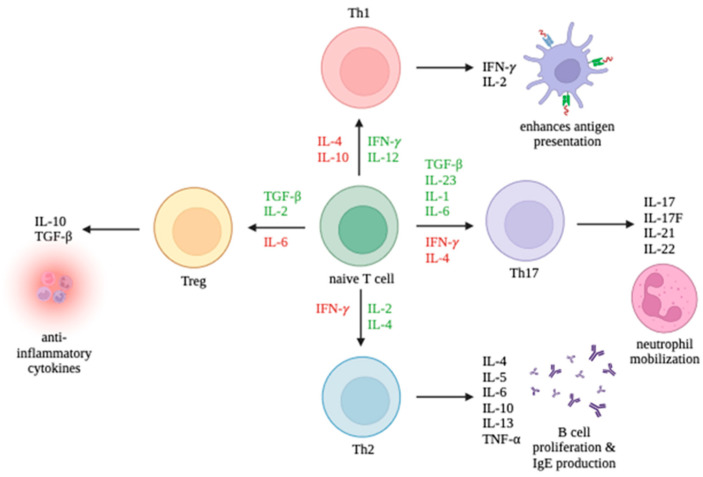
Differentiation of T cell subsets Th1, Th2, Th17, and Tregs and the production of their respective cytokines. Cytokines promoting differentiation toward a cell type are in green and cytokines inhibiting differentiation are in red.

CD4+ T cells can differentiate into multiple lineages of Th cells, including Th1, Th2, Th17, and Treg, depending on the cytokines found in their environment. The promotion and inhibition of these lineages are summarized in Figure 1. Th1 cells primarily enhance antigen presentation. Th2 cells promote B cell proliferation and IgE production. Th17 cells mobilize neutrophils. Treg cells release anti-inflammatory cytokines. While Th1 and Th17 cells can trigger autoimmune responses, Treg cells can suppress autoimmune responses. It is the interplay between the T cell immune response and viruses which largely determines the outcome of an infection. 

Although there is no complete and reliable model to explain the progressive depletion of CD4+ T cells following HIV infection, evidence supports an interplay between direct impacts of viral replication, chronic inflammation as an indirect effect of viral replication, and host immune system dysregulation [11].

**Figure 2:**
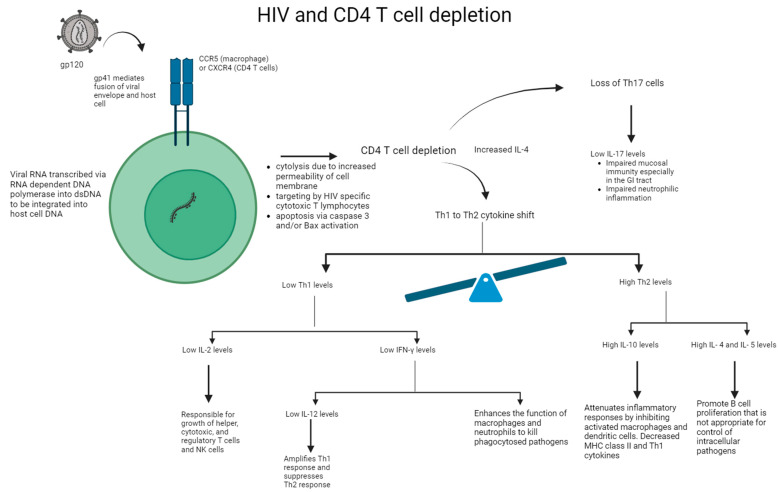
Mechanism of HIV on CD4+ T cell depletion and impaired immune response. HIV binds to the CD4+ protein on the cell surface of T cell or macrophage via its gp120 envelope protein. The virion gp120 protein then interacts with a chemokine receptor: CCR5 of macrophages or CXCR4 of CD4+ T cells. Gp41 is inserted into the host cell leading to CD4+ T cell depletion and viral replication. Following CD4+ T cell depletion, IL-4 is increased, leading to the Th1 to Th2 cytokine shift and loss of Th17 cells. Overall, there is a reduction in IL-2, IFN-γ, IL-12, and IL-17 levels. In addition, there is an increase in IL-4, IL-5, and IL-10 levels, thereby leading to impaired immune response.

In the acute phase of HIV infection, the direct impact of viral replication has been shown to significantly contribute to the initial loss of CD4+ T cells via: (i) the increased permeability of the cell membrane after viral budding and/or syncytium formation resulting in cytolysis, (ii) the induction of HIV-specific CD8+ cytotoxic T lymphocytes, which target and eliminate virally infected cells, and (iii) the programmed cell death of cells undergoing productive infection due to caspase-3 and/or Bax activation [11]. See Figure 2. After this acute phase is over and host CD4+ memory T cells undergo a temporary phase of regeneration, patients with HIV experience another progressive decline in CD4+ T cell counts. Several mechanisms have been proposed to explain this: (i) bystander apoptosis occurs due to viral proteins promoting the apoptosis of nearby cells (notably HIV-1 gp120 after its interactions with CD4+ and CCR5 or CXCR4 coreceptors), (ii) immune activation induces FasL production and Fas (CD95) expression in nearby, uninfected CD4+ T cells, resulting in shortened lifespans and an increased sensibility to activation-induced cell death (AICD), and (iii) abortive infection leading to pyroptosis through the caspase-1 pathway, due to an accumulation of incomplete reverse transcripts and the induction of antiviral and inflammatory responses [11]. 

Although the overall number of CD4+ T cells can initially regenerate, different subsets of CD4+ T cells (i.e., Th1, Th2, Th17, Treg, etc.) are disproportionately depleted and never return to homeostatic levels [12]. Since each T cell lineage has a specialized immunological function, the impact of HIV infection is a disrupted and often overly robust inflammatory response to antigens an with impaired maintenance of these T-cell subsets.

## 4. HIV’s Effect on Inflammation

A key characteristic of HIV infection is the persistence of chronic immune activation and inflammation which is seen throughout the course of infection, even when viral load is decreased to an undetectable level. This aberrant immune activation and inflammation is understood to be a significant driver of CD4+ T-cell depletion and increased susceptibility to opportunistic pathogens. Several mechanisms have been proposed to help explain this complex process, including (i) the persistence of HIV viral reservoirs, (ii) the dysregulation of intestinal mucosa and microbial translocation, (iii) the depletion of regulatory T (Treg) cells, and (iv) co-infection with other viruses [13].

Patients in the acute and chronic stages of HIV infections have been shown to have elevated pro-inflammatory cytokines, such as TNF-α, IL-6, and IL-17 [14]. In Individuals with chronic HIV, elevated TNF-α concentrations indicate long-term inflammation [15]. 

Additional data have shown a correlation between diminished CD4+ T cell counts and increased levels of pro-inflammatory cytokines (IL-1β, IL-17, and IL-6) that contribute to systemic inflammation, and malondialdehyde (MDA), which is an end-product of lipid-peroxidation and a marker for oxidative stress. When plasma samples of Individuals with HIV with low CD4+ T cell counts < 200 cells/mm^3^ and those with T cell counts of 200–300 cells/mm^3^ were measured, concentrations of IL-1β, IL-17, IL-6, and MDA were observed to be elevated [16]. The high levels of oxidative stress seen in these chronic inflammatory states requires a response from the body’s antioxidants, of which glutathione is the most abundant. Unfortunately, by mechanisms explained in the next section, glutathione levels are low in patients with HIV.

In HIV-seropositive individuals, there is a shift in CD4+ T helper (Th) cell responses from Th1 to Th2 [17]. Unfortunately, Th2 response alone does not adequately control the intracellular pathogens and antibodies seen in HIV, and patients are unable to neutralize the virus. As a result, the virus persists and spreads in CD4+ T cells leading to a loss of immunity and susceptibility to opportunistic infections.

HIV also leads to the chronic overproduction of the anti-inflammatory profibrotic cytokine TGF-β, which, paradoxically, is a major cause of immunosuppression in HIV infection. An overproduction of TGF-β has been shown to suppress the host immune response by directly targeting cells of adaptive and innate immunity. TGF-β’s profibrotic activity also indirectly decreases T cell production in secondary lymphoid tissues and compromises the repopulation of T-cell dependent zones. This process is believed to contribute to the progression of HIV into AIDS and to the overall pathogenesis of diseases associated with HIV infections [18]. 

Our lab has found that the cytokine imbalance and immunological dysregulation associated with HIV infection may be partially restored with the use of glutathione (GSH) [10]. When a liposomal glutathione (L-GSH) supplement was given to Individuals with HIV with low CD4+ T cell counts, below 350 cells/mm^3^, over a 3-month period, there was a notable increase in levels of IL-2, IL-12, and IFN-γ, with a concomitant decrease in levels of IL-6, IL-10, and free radicals, and stabilization in the levels of TGF-β, IL-1, and IL-17, compared to their placebo counterparts [19]. These findings suggest a restorative benefit of L-GSH supplementation in redox homeostasis and cytokine balance in HIV-positive patients. 

## 5. Glutathione: Inflammation and Immune Response

Glutathione (GSH) is a tripeptide antioxidant important in the maintenance of cellular redox states and cellular homeostasis. It is present in all mammals and primarily synthesized within the liver to be stored in the cytosol and organelles of animal cells. The protective effects of GSH include the neutralization of oxygen radicals, the maintenance of mitochondria and mitochondrial DNA, the chelation of heavy metals such as mercury, the regeneration of vitamins C and E, the regulation of apoptotic pathways, and action as a cofactor for antioxidant enzymes [20]. Although GSH is not alone responsible for these protective mechanisms, the hepatic production of GSH seems to be essential to survival, with knockout mice dying within months secondary to inflammation and hepatic steatosis resulting in liver failure [21]. The negative implications of decreased GSH tend to revolve around inflammation and impaired antioxidant capabilities, especially in the mucosal cells of the gut where they are most heavily stored [22]. For these reasons, understanding GSH and its effects on the body in both adequate and inadequate supplies remains important.

**Figure 3:**
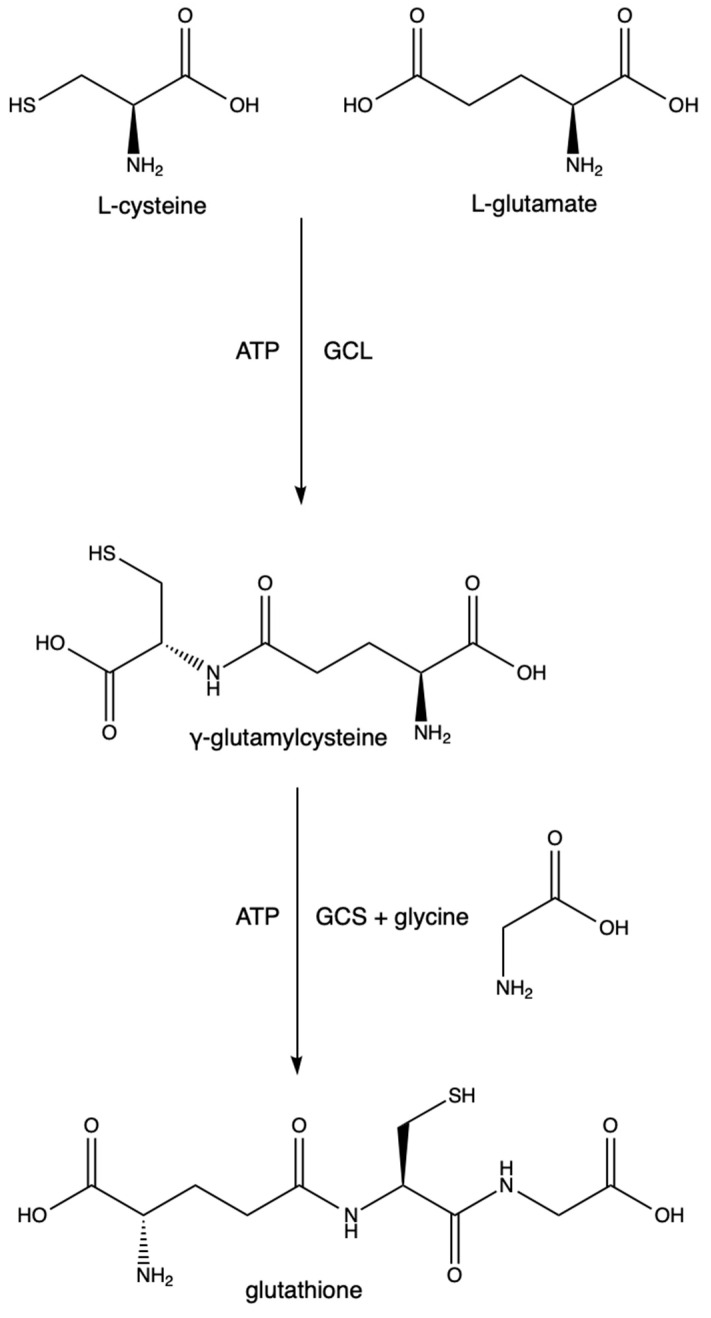
Glutathione synthesis.

The synthesis of GSH is a two-step process depending on cysteine and glutamate as substrates. The first step is the rate-limiting step and is driven by the enzyme glutamate cysteine ligase (GCL) (previously known as γ-glutamylcysteine synthetase and γ-glutamylcysteine synthase). It catalyzes the conversion of L-cysteine, L-glutamate, and ATP into γ-glutamylcysteine. GCL is made of two subunits (GCLC (a catalytic subunit) and GCLM (a modifier subunit)). GCLC contains the active site in which the L-cysteine binds to the γ-carbon of L-glutamate. GCLM is responsible for lowering the Km of the reaction and increasing GCL’s affinity for glutamate and ATP, thus making the reaction more efficient [23]. Mutations in either of these subunits are points of polymorphisms seen at least in schizophrenia [24], stroke [25], diabetes [26], asthma [27], MI [28], and coronary artery disease [29]. The excessive synthesis of GSH results in increased melanoma growth and increased resistance to some cancer drugs and radiation therapies [30]. The second step utilizes GSH synthetase, an enzyme responsible for forming GSH from glycine and γ-glutamylcysteine. See Figure 3.

**Figure 4:**
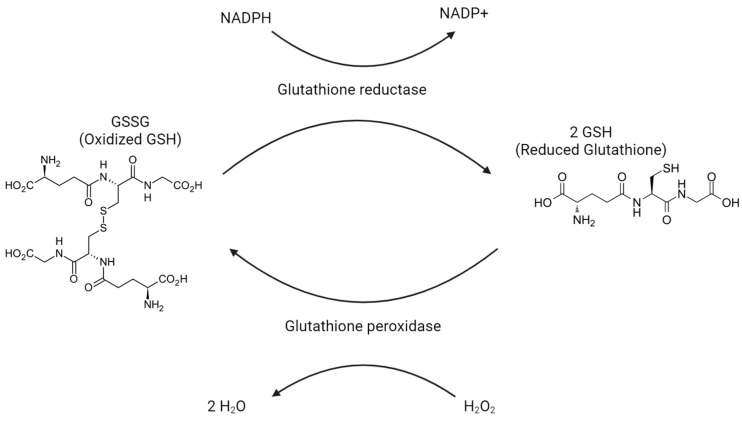
Glutathione peroxidase oxidizes H_2_O_2_ into H_2_O, as oxidized glutathione (GSSG) is reduced to reduced glutathione (GSH).

Glutathione exists in one of two states: a reduced form called GSH and an oxidized form called GSSG. One of the main responsibilities of glutathione is the reduction of H_2_O_2_ into H_2_O as GSH is oxidized into GSSG. See Figure 4. This conversion from H_2_O_2_ into H_2_O prevents H_2_O_2_ from turning into harmful oxygen radicals. In most of the body, the enzyme that carries out this reaction is glutathione peroxidase. Within peroxisomes, the required enzyme is peroxidase. In order to replenish GSH, GSSG is reduced back to GSH by glutathione reductase. In cases in which GSSG is not able to be reduced quickly enough, GSSG can be exported via protein sulfhydryl groups. GSH will largely be found in the reduced/GSH state, stored 80–85% in the cytosol, 10–15% in mitochondria, and another 1% in the endoplasmic reticulum. GSH eventually breaks down into cysteine, glutamate, and glycine in the plasma and bile to be used elsewhere.

Adequate levels of reduced GSH are also maintained indirectly by the enzyme glucose 6 phosphate dehydrogenase (G6PDH) since G6PDH is involved in producing NADPH. Viral illnesses reduce G6PDH levels, which seems to be at least one mechanism behind the decreased GSH levels seen in patients with HIV. 

Since GSH oxidation involves hydrogen peroxide reduction, any issue in GSH metabolism is apt to cause an overabundance of oxygen radicals. If there is poor GSH synthesis due to low cysteine levels, inadequate GCL enzyme, inadequate GSH synthetase, or mutations in GCLC or GCLM, then we can expect to see problems in the reduction of peroxides and a resultant increase in free oxygen radicals. Since the synthesis of GSH involves the unique peptide bond on a γ-carbon, the enzyme γ-GGT is also necessary in order for GSH to be hydrolyzed. γ-GGT deficiency will thus result in oxidative stress [31]. 

In addition to excessive amounts of free radicals found when GSH is low, we see excessive inflammation marked by increased levels of IL-1, IL-6, and TNF-α [9]. The increase in pro-inflammatory cytokines reciprocally amplifies the production of oxygen radicals, which are scavenged by free GSH, creating a cycle where low GSH levels contribute to both increased inflammation and free radical production. 

Low levels of GSH also correlate with a compromised immune response by altering cytokine production, where decreased GSH is associated with reduced levels of IL-12, IL-2, and IFN-γ, which play pivotal roles in immune regulation. Specifically, reduced IL-12 hampers Th1 cell differentiation and macrophage activation, reduced IL-2 compromises T-cell proliferation, and reduced IFN-γ impairs intracellular defense [19]. Consequently, diminished GSH undermines the coordinated immune response, disrupting cell-mediated immunity and weakening the ability to combat infections.

Patients infected with HIV exhibit diminished levels of GSH in plasma, erythrocytes, lymphocytes, and peripheral blood mononuclear cells (PBMCs), which is largely attributed to the enhanced inflammation and ROS production caused by the virus, and this GSH deficiency has been correlated with the dysregulated Th1-to-Th2 shift in immune response [4,32]. As mentioned, GSH plays a crucial role in modulating the function of various immune cells, and its deficiency emerges as a significant factor contributing to immune dysfunction in Individuals with HIV. While GSH depletion and the disruption of redox homeostasis has been observed in other viral infections, such as IAV and HSV-1, the most extensive data concerning GSH homeostasis alteration is documented in HIV infection [33]. Inadequate GSH levels compromise immune cell activities, leading to weakened phagocytosis by macrophages, diminished cytotoxicity in NK cells, impaired antigen presentation by dendritic cells, and compromised proliferation and signaling in lymphocytes. Overall, the depletion of GSH in Individuals with HIV emerges as a critical factor disrupting redox homeostasis, jeopardizing the intricate coordination of immune cell activities, and contributing to immune dysfunction, rendering them more susceptible to secondary infections, particularly Mycobacterium tuberculosis (TB) [34,35].

## 6. Glutathione: Mechanisms

The effects of GSH on cytokine levels have been well studied, but the mechanisms and pathways connecting GSH to these effects have been reviewed much less. With each attempt at using GSH to treat a disease, one angle of the mechanism is inspected a little bit more. It is the goal of this section to review some of the mechanisms that have been more clearly illuminated through clinical trials involving GSH.

It is well known that HIV causes shifts in T helper cell lineages, with the most cited and well understood shift involving Th1 and Th2 cells. There has not been as much work relaying the effects of GSH on Treg cells, yet one group was able to unveil much of the potential mechanism through a series of trials conducted on mice. Mice were given RSL3, a GSH peroxidase 4 (Gpx4) inhibitor, and found that Tregs were not able to survive minimal amounts of lipid peroxidation [36]. RSL3-treated Treg cells accumulated lipid peroxides and died through a resultant ferroptosis, suggesting that Gpx4 is integral to maintaining Treg cell homeostasis. The same group further reported significant increases in IL-17 from Th17 cells in mice with floxed Gpx4 alleles, suggesting that the proper homeostasis of GSH through Gpx4 specifically is relevant to the homeostasis of multiple T helper cell lineages.

When lipopolysaccharide (LPS) is presented to the immune system, the response is to activate cells capable of destroying the Gram-negative pathogen. This response includes the activation and localization of neutrophils which play their role by utilizing their respiratory burst pathways in which oxygen is converted to a singlet oxygen and then to a hydrogen peroxide molecule and hypochlorite which results in death of the bacteria. All of these reactive oxygen species trigger NFκΒ, a transcription factor highly sensitive to oxidative stress. NFκB then triggers other cells to release cytokines. In alcoholic liver disease, we see NFκB triggering monocytes and Kupffer cells in the hepatic sinusoids to release IL-6, IL-8, and TNF. Giving GSH to these individuals resulted in the decreased activity of NFκB in vitro and in their resultant cytokines in vivo [37,38]. Other studies illuminate a more direct role of NFκB in which one of its binding elements acts as a necessary activator for the promoter region of the IL-6 gene [39]. 

Oxidative stress can also be caused by high ACE:ACE2 ratios. Such is the case in individuals with SARS-CoV-2, which downregulates ACE2 on cell surfaces [40]. Inflammation here is a result of an overactive renin angiotensin system. GSH plays a helpful modulatory role in this situation by decreasing ACE activity in the reduced state of GSH (rGSH) and increasing it in the oxidized state (GSSG) [41]. This fits the pattern of rGSH playing an anti-inflammatory role, with the expected improvement of any inflammatory process in individuals who receive glutathione in the reduced form (the form given in treatments). One of such diseases is HIV.

In patients with AIDS and TB, patients receiving N-acetylcysteine (NAC), a precursor to GSH, had increased GSH levels, increased IFN-γ levels, and decreased IL-1, IL-6, and TNF-α [42]. Although this study did not measure any intermediate markers between GSH and the resultant cytokine changes, the pattern of change in cytokine levels suggests decreased macrophagic response to antigens, as IL-1, IL-6, and TNF-α are released primarily by macrophages. We know that at least some macrophagic activity depends on GSH, with mutant strains of *Mtb* lacking small peptide transporters resulting in increased survivability compared to *Mtb* without the mutation [43]. The lack of small peptide transporters resulted in nitric oxide being unable to enter and damage *Mycobacterium* cells via the small peptide S-nitrosoglutathione.

## 7. Glutathione: Treating HIV- and AIDS-Related Diseases

The previous mechanisms show a common thread of GSH improving inflammatory states through a variety of macrophages and neutrophils, most of which involve an improvement in the reactive oxygen or reactive nitrogen species pathways. We also see resultant declines in pro-inflammatory cytokines and an increased activation of anti-inflammatory cytokine production. In addition, GSH is correlated with the apoptosis of many cell lines, including CD4+ T cells, although the causal relationship is less well understood [44]. It is through these mechanisms and trials that we continue to investigate GSH use in treating HIV patients, as the very same reactive oxygen and nitrogen pathways and imbalances in inflammation are drivers of the disease’s immunodeficient states.

### 7.1. Effects of Glutathione on Metabolic Health and Overall Survival in Patients with HIV

HIV-positive patients demonstrate decreased levels of serum GSH and glutathione precursors, including cystine and glycine, which can lead to impaired cell survival, and further the progression of the disease [45].

A 1997 study showed that subjects with CD4+ T cell counts below 200 cells/mm^3^ had lower GSH levels compared to subjects measured in earlier stages of HIV disease [46]. Additionally, survival analyses revealed a close relationship between levels of GSH -S-bimane fluorescence (GSB), a measure of intracellular GSH, and survival. Subjects with higher GSB levels tended to survive longer than subjects with lower GSB levels. Furthermore, subjects with the lowest GSB levels were those with CD4+ T cell counts below 200 cells/mm^3^.

Preliminary evidence from this paper also suggested oral administration of NAC could replenish GSH levels and potentially improve the subjects’ survival. Subjects given oral NAC for 8 weeks demonstrated statistically significant restorations of whole blood GSH levels compared to their placebo counterparts [46]. A majority of subjects enrolled in the study were able to either continue or start NAC supplementation for 6 months, with results showing increased survival. The research also hypothesized that precautions could be taken to prevent further GSH deficiency such as decreasing alcohol intake, or medications containing acetaminophen, as well as avoiding excessive UV irradiation exposure in order to improve HIV-patient survival. 

Reduced glutathione levels are associated with oxidative damage to many organs, including the heart, kidneys, and liver. Oxidative damage can be spurred on by disease, recreational drug use, alcohol use, medications, and many other processes. It is not hard to extrapolate, then, that patients with multiple contributing factors for reduced glutathione levels may experience more severe disability and worse outcomes due to the loss of the antioxidant. For example, in patients with HIV, chronic alcohol use leads to exaggerated oxidative damage to plasma DNA compared to those who do not consume alcohol [47]. For this reason, maintaining GSH seems to be integral to metabolic health, especially in patients with HIV. 

In 2017, our lab conducted a study in which 14 HIV patients with CD4+ counts less than 350 cells/mm^3^ were treated with liposomal glutathione (L-GSH from Your Energy Systems) for 3 months [19]. Prior to treatment, the patients had low levels of IL-2, IL-12, and IFN-γ and high levels of IL-6, IL-10, and TGF-β. After the 3-month trial, the group receiving L-GSH had increased levels of IL-2, IL-12, and IFN-γ and decreased levels of IL-6, IL-10, and TGF-β nearing (and in some cases surpassing) individuals without HIV. Our lab conducted a similar study in 2015 in which 15 HIV patients without chronic disease were treated with L-GSH for 13 weeks, leading to the restoration of Th1 cytokine responses and statistically significant increases in IL-1β, IL-12, IFN-γ, and TNF-α [10]. In addition, the results showed decreases in the levels of free radicals, IL-10, and TGF-β compared to counterparts receiving a placebo.

HIV-positive patients also demonstrate metabolism defects related to the progression of HIV, including increased oxidative stress, insulin resistance, mitochondrial dysfunction, cognitive defects, inflammation, and endothelial dysfunction, etc. In 2020, a clinical trial with eight HIV-positive patients was monitored after the supplementation of GlyNAC capsules containing glycine (1.31 mmol/kg/d) and NAC (0.83 mmol/kg/d) for a 12-week period. Patients were assessed again 8 weeks after discontinuing GlyNAC supplementation. Oxidative stress was measured using a thiobarbituric acid-reduction substance assay (TBARS) and an Iso-Prostaglandin-F2α assay. After the 12-week period, measurements of muscle GSH concentration increased by 340%, while TBARS decreased by 81%, and F2-isopropanes decreased by 80%, meaning decreased oxidative stress [48]. Other findings include decreased insulin resistance by 69%, improvements in cognitive function, a decreased plasma concentration of inflammation biomarkers (IL-6, hsCRP, TNFα), endothelial dysfunction (E-selectin, SICAM1, sVCAM1), and a decline in genomic damage. As for mitochondrial dysfunction, GlyNAC supplementation improved mitochondrial fuel oxidation and lowered mitochondrial glucose oxidation. Physical function, such as forearm grip strength and gait speed, were also improved. These outcomes receded after GlyNAC supplementation was discontinued. The results of this study encourage the use of GlyNAC in patients with HIV to prevent defects related to GSH deficiency. Further clinical testing is required to assess the long-term effects of GlyNAC supplementation, and possible withdrawal effects.

### 7.2. Effects of Glutathione on HIV-Associated Neurodegenerative Diseases 

HIV-associated neurodegenerative diseases (HAND) encompass a spectrum of progressive cognitive and neurologic symptoms such as deficits in memory, attention, and concentration [49]. Many of these diseases involve a significant inflammatory component in which C-C motif chemokine ligand 2 (CCL2), a chemokine secreted by monocytes, macrophages, and dendritic cells, attracts monocytes, T cells, and other leukocytes to a site of acute infection [50]. HIV leads to the dysregulated binding of CCL2 to its receptor, C-C motif chemokine receptor 2 (CCR2), on neurons, astrocytes, and brain microvascular endothelial cells, ultimately leading to the increased permeability of the blood–brain barrier (BBB) [51]. 

HIV-infected leukocytes can then infect the healthy brain that would have been otherwise blocked by the BBB and begin to directly cause neuronal dysfunction by inducing chronic inflammation or neuronal degeneration via Gp120, Tat, and VPR proteins [4]. As a result of increased CCL2 levels, patients are more susceptible to developing HIV encephalitis and HIV-1-associated dementia [4]. Research shows that patients with HAND demonstrated higher gp120 and Tat proteins, which induces ROS production. The increased oxidative stress then leads to decreased cell viability, increased lipid peroxidation, and increased ceramide production, which are hallmarks of HIV-related dementia [52]. 

By introducing NAC, patients with HIV demonstrated lower TNF levels and a reduction in the rate of decline of CD4+ T cell counts [53]. In addition, oxidative stress was reversed, thus preventing the neurotoxicity prevalent in patients with HIV and AIDS [54]. NAC injected into rodents increased GSH levels in the brain, therefore protecting the brain from radical oxidative stress and lipid peroxidation [55]. With this evidence in mind, GSH can assist not only in preventing the progression of HIV but also in preventing the progression of HAND. 

### 7.3. Effects of Glutathione on HIV and Th17 Depletion in GI Tract 

In the GI tract, Th17 cells maintain mucosal integrity by producing IL-17, which is a cytokine responsible for mobilizing neutrophils and myeloid cells and also for regeneration of epithelial cells via claudin induction [56]. Th17 cells are also responsible for producing IL-22, which maintains intestinal integrity and contributes to bacterial clearance. While Th17 cells can respond appropriately to bacterial and fungal antigens such as *Staphylococcus aureus* and *Candida albicans*, their immune responses are not specific to viral antigens [57]. As a result, Th17 cells are incapable of containing HIV infections on their own. 

A 2008 study demonstrated a significant decrease in Th17 cells in the GI tract of patients with HIV compared with people without HIV [58]. Although the mechanism that leads to the depletion of Th17 cells remains largely unknown, some of the proposed mechanisms include (i) a lack of HIV-inhibitory RNases in human Th17 cells leading to early, sustained depletion [59], (ii) decreased levels of IL-21-producing cells responsible for maintaining Th17 cells [60], and (iii) a depletion of CD103+ dendritic cells which promote Th17 differentiation [61]. As a result, the impaired Th17 responses in individuals with HIV lead to increased susceptibility to opportunistic diseases including tuberculosis and candidiasis [62,63]. In addition, depleted Th17 cells result in epithelial dysfunction in the GI mucosa, leading to more general gastrointestinal issues including fat malabsorption, bacterial overgrowth, and HIV-related diarrhea [64]. Overall, patients with HIV demonstrate a preferential loss of Th17 cells, leading to increased susceptibility to opportunistic co-infections and compromised gut mucosal defenses against bacterial and fungal infections. 

While there is minimal research to conclude that GSH can improve Th17 activity in the context of HIV, there is a 2023 study that concludes that GSH is crucial in maintaining IL-17 and IL-22 levels via Th17 cells during an acute infection [65]. Mice that underwent GCLC ablation were found to have lower levels of GSH and a severely impaired Th17 immune response to GI infections demonstrating decreased survivability compared to non-ablated counterparts [65]. For this reason, GSH may serve an additional role in maintaining the integrity of the intestinal barrier and protecting HIV-positive patients from opportunistic diseases and gastrointestinal inflammation.

### 7.4. Effects of Glutathione on HIV and Tuberculosis 

Patients coinfected with HIV and TB are typically treated with both anti-tubercular and antiretroviral agents. The co-administration of drugs reduces mortality but also introduces more drug–drug interactions and increases the risk of developing immune reconstitution inflammatory syndrome (IRIS) [66]. IRIS is a state of upregulated immune responses seen in up to one third of individuals starting highly active antiretroviral therapies (HAART). CD4+ T cell counts begin to rise as expected in response to therapy, but patients who develop IRIS will additionally experience an excessive inflammatory response to prior infections, typically within 6 months of initiating HAART. While the majority of patients on HAART demonstrate improved mortality rates, patients with IRIS develop new clinical presentations associated with the organism involved, therefore leading to significant adverse outcomes. For example, patients on HAART coinfected with HIV and TB may develop tuberculous lymphadenitis, cutaneous lesions, peritonitis, epididymitis, bowel perforations, and granulomatous nephritis after developing IRIS [67]. 

Individuals with HIV are more likely to contract tuberculosis due to the decreased GSH levels which further lead to decreased Th1 cytokines [68]. In a previous paper, we proposed that GSH supplementation may mitigate the symptoms of TB in HIV-positive patients because of this decline in GSH [69]. One study found that treating subjects with HIV with NAC, a GSH precursor, inhibited the growth of *Mycobacterium tuberculosis (Mtb)* [42]. This was evidenced by decreased levels of IL-1, TNF-α, and IL-6, and increased levels of IFN-γ which led to an activated macrophagic response that restricted *Mtb* replication [70]. There are no concrete answers regarding GSH as a therapy option for the management of IRIS at the moment. However, a 2023 study followed 316 patients in China over 12 weeks of HAART therapy and found increases in malondialdehyde (MDA) and Th17 cell counts and decreases in superoxide dismutase (SOD) and Treg levels [71]. This constellation of findings indicates that GSH supplementation may be a viable treatment option for patients with IRIS or as a prophylactic agent in patients starting HAART.

## 8. Conclusions

This literature review investigates the potential role of GSH on the cell-mediated immune responses in HIV and HIV-associated diseases. While antiretroviral therapies have long been the primary treatment to help prevent the progression of HIV to AIDS, research has shown that GSH also serves as an effective adjunctive therapy for patients with HIV. GSH is cheaper to produce than antiretrovirals and may serve as a viable treatment option for those previously on limited or no therapy due to cost. This is especially relevant considering that most patients with HIV live in Africa where supplies can be costly and limited. Patients with HIV demonstrate both a reduced quantity and efficacy of CD4+ T cell response and increased inflammatory cytokine levels. Thus, patients with HIV have impaired cell-mediated immunity and increased susceptibility to other deadly opportunistic diseases such as TB, candidiasis, and neurodegenerative diseases. GSH, an essential antioxidant in the body, is shown to be depleted in HIV-seropositive patients. Supplementing patients with L-GSH has been shown to increase IL-2, IL-12, and IFN-γ and decrease IL-6, IL-10, and TGF-β, thus resulting in an overall more appropriately balanced immune response to pathogens. Furthermore, GSH supplementation has been shown to protect HIV patients from a variety of opportunistic diseases by (i) mitigating oxidative stress and chronic inflammation in HIV-associated neurodegenerative diseases, (ii) improving Th17 responses in HIV patients who have impaired gut mucosal defenses, and (iii) restoring Th1 responses to protect patients from TB coinfection. Further research can be focused on understanding GSH’s potential role in restoring Th17 responses specific to HIV-associated GI complications and its impact on the development of IRIS if co-administered with antiretroviral agents. By better understanding GSH’s mechanism and effects, we may be able to make stronger recommendations for GSH supplementation as a targeted adjunct or solo therapy option in various patient subgroups.

## Data Availability

Not applicable.
